# Continuing Professional Development – Medical Imaging

**DOI:** 10.1002/jmrs.644

**Published:** 2023-01-10

Maximise your CPD by reading the following selected article and answer the five questions. Please remember to self‐claim your CPD and retain your supporting evidence. Answers will be available via the QR code and online at www.asmirt.org/news-and-publications/jmrs, as well as published in JMRS – Volume 70, Issue 4, December 2023.

## Medical Imaging – Original Article

### Exploring the role of medical imaging assistants in Australian medical imaging departments: a mixed‐methods study

Pinson JA, King OA, Dennett AM, Davis A, Williams CM, Snowdon DA. (2023) *J Med Radiat Sci*. 10.1002/jmrs.623

1. In this study, what was the most common communication mode between colleagues and patients?
  - Phone
  - Electronic
  - Face‐to‐face
  - Communication book
2. Which task did medical imaging assistants spend the most time doing?
  - Moving equipment
  - Transferring patients
  - Facilitating patient preparation
  - Managing the booking schedule
3. How many different tasks did participants identify in this study?
  - 21
  - 23
  - 27
  - 32
4. What was the total time motion data collected?
  - 1470 min
  - 4170 min
  - 4710 min
  - 7140 min
5. What was one of the three major themes identified in this study?
  - Tasks are important to workflow
  - Tasks are predominantly patient facing
  - Medical imaging assistants love their job
  - Medical imaging assistants communicate mostly with medical imaging professionals

### Recommended further reading:

1. Snowdon DA, King OA, Dennett A, Pinson JA, Shannon MM, Collyer TA, Davis A, Williams CM. Delegation of patient related tasks to allied health assistants: a time motion study. *BMC Health Serv Res* 2022; 22(1): 1280.
2. King OA, Pinson JA, Dennett A, Williams C, Davis A, Snowdon DA. Allied health assistants' perspectives of their role in healthcare settings: a qualitative study. *Health Soc Care Community* 2022. 10.1111/hsc.13874
3. Cartwright AK, Pain T, Heslop DJ. Substitution, delegation or addition? Implications of workforce skill mix on efficiency and interruptions in computed tomography. *Aust Health Rev* 2021; 45(3): 382‐8.

## Answers


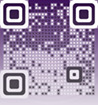


Scan this QR code to find the answers, or visit www.asmirt.org/news-and-publications/jmrs

